# Functional roles of the thalamus for language capacities

**DOI:** 10.3389/fnsys.2013.00032

**Published:** 2013-07-16

**Authors:** Fabian Klostermann, Lea K. Krugel, Felicitas Ehlen

**Affiliations:** Department of Neurology, Motor and Cognition Group, Charité - University Medicine BerlinCBF, Berlin, Germany

**Keywords:** thalamus, language, corrtex, selective engagement, basal ganglia

## Abstract

Early biological concepts of language were predominantly corticocentric, but over the last decades biolinguistic research, equipped with new technical possibilities, has drastically changed this view. To date, connectionist models, conceiving linguistic skills as corticobasal network activities, dominate our understanding of the neural basis of language. However, beyond the notion of an involvement of the thalamus and, in most cases also, the basal ganglia (BG) in linguistic operations, specific functions of the respective depth structures mostly remain rather controversial. In this review, some of these issues shall be discussed, particularly the functional configuration of basal network components and the language specificity of subcortical supporting activity. Arguments will be provided for a primarily cortico-thalamic language network. In this view, the thalamus does not engage in proper linguistic operations, but rather acts as a central monitor for language-specific cortical activities, supported by the BG in both perceptual and productive language execution.

## Introduction

Over the last decades, functional imaging and neurophysiological techniques have allowed for an increasingly detailed allocation of mental functions to cortical structures and processes, whereas subcortical functions in cognitive processing still remain somewhat elusive. This discrepancy might, amongst others, be due to the fact that a precise assessment of processes in tiny and remote depth structures is comparably difficult with the prevailing research tools, such as magnetic resonance imaging (MRI) or electroencephalography (EEG). Thus, methodological properties could to a certain extent reinforce classical cortico-centric concepts of cognitive and, particularly, language capacities, based on the fundamental findings of Broca and Wernicke in the nineteenth century (Broca, 1861; Wernicke, 1874).

It is not a new claim, however, that a structural requisite of complex behavioral functions is the evolution of specific two-way thalamo-cortical operational units (Sanides, 1970). Concerning this view, corroborating information appears to come in from clinical observations in the many patients who, alongside with thalamic lesions, develop cognitive dysfunctions. Respective deficits can affect a wide range of behaviors, related to perceptual, attentional, mnemonic, executive or, specifically, linguistic capacities (e.g., Dejerine and Roussy, 1906; Bogousslavsky et al., 1988; Van Der Werf et al., 2000; Fimm et al., 2001; Liebermann et al., 2013), but naturally this notion leaves many questions open. For example, do thalamic structures take part in the proper programming of behaviors? Or do they rather ‘just’ relay information between cortical regions involved in the generation of given mental actions? Are relay functions stable or flexible, and how should flexibility be implemented? Finally, can ‘thalamic functions’ be generalized, or are they specific for any given task or process?

These questions shall be shortly discussed against the background of concepts of thalamo-cortical working models, ideas on thalamic functions in cognition and respective clinical data in the particular field of human language.

## Basic principles of thalamo-cortical interaction

Growing insight into principles of cortico-thalamic communication let the label ‘cortical function’ for the designation of higher-order processes appear out-dated. The basic idea underlying this term was that, once primary, e.g., sensory, information has reached its cortical projection area via thalamic relay nuclei, the finishing of respective processing would comprise a chain of exclusively cortical processes. This view, however, seems in contradiction to more recent descriptions of cortico-thalamic signaling (e.g., Murphy et al., 1999; Sherman and Guillery, 2011). In modern concepts, the thalamus consists of first-order nuclei which relay mainly sensory inputs to their cortical projection areas, and, to a larger extent, of higher-order nuclei which propagate information from one cortical area to another and also receive messages from their projection sites. In so doing, a number of compositional and cellular properties entail that thalamic nuclei *ab initio* act on the messages to and from cortical areas (Guillery and Sherman, 2002).

Even in first-order nuclei, such as the lateral geniculate, only a minority of synaptic connections comes from the retina; the vast majority of contacts is made with modulatory cells, e.g., local interneurons or layer VI and, in case of higher order thalamic nuclei, also layer V corticofugal neurons (Van Horn et al., 2000; Lam and Sherman, 2010). With respect to layer V output, it appears further interesting that, while having another target projection, e.g., on spinal or brain stem motor neurons, the connection with the thalamic neuron derives from an axonal branch. Thus, higher-order nuclei seem to be informed about output signals (transformed into behavior) by efference copy which, in turn, acts on the information sent back from the thalamus to the subsequent cortical projection (cf. Von Holst and Mittelstaedt, 1950; Sherman, 2005).

Besides the description of this connectivity, meticulous research has demonstrated that thalamic relay neurons modulate informational transfer by working in specific discharge modes (cf. Jahnsen and Llinas, 1984; Steriade et al., 1998; Steriade, 2000). Under the condition of relatively depolarized membrane potentials prevailing in awake state, cells convey synaptic input in a tonic ‘spiking mode’, supposed to be the basis of relatively precise, i.e., ‘linear’ transmission of presynaptic to postsynaptic action potentials in a fairly wide frequency range (McCormick and Feeser, 1990). This functional state can, at least regarding first-order thalamic nuclei, be conceived as a prerequisite for providing the organism with exact information about its physical environment, but also about internal or even mental states. Further, with relatively hyperpolarized membrane potentials thalamic relay neurons may discharge so called low-threshold calcium spikes, triggering bursts of action potentials. During sleep, this bursting mode, which only occasionally occurs in the awake state, becomes rhythmic and entrains larger assemblies of neighboring neurons. This propensity is only interrupted by relatively strong stimuli, so that it disconnects the organism from real world information (McCormick and Feeser, 1990). While awake, for reasons of membrane and synaptic physiology, bursting of relay neurons is usually followed by tonic spiking. In this case, the initial burst discharged from the thalamus has been proposed to act as a ‘wake-up call’ to get the cortex engaged in the processing of the subsequently conveyed information (Guido and Weyand, 1995; Sherman, 2001; Swadlow and Gusev, 2001; Swadlow et al., 2005). The control of these states is not fully understood, but certainly the reticular nucleus, surrounding the thalamus as a thin cell sheet of GABA-ergic inhibitory neurons comes into play here. The reticular neurons are both driven by branches from ascending relay neurons and related corticofugal output from layer VI, and hyperpolarise the driving thalamo-cortical cells via back-projections. Thus, the control of the discharge mode and thalamocortical transmission, appears to be organized in a dynamic balance of bottom-up and top–down factors (Yingling and Skinner, 1977; Crick, 1984; Guillery et al., 1998; Lam and Sherman, 2010).

Altogether, the properties of thalamic relay together with reticular neurons seem to serve a flexible spatiotemporal gating of information on the way to and between cortical areas. Distinct discharge modes allow for recruiting downstream targets or disrupting them from further informational flow. This and their widespread connections appear to enable thalamic nuclei to monitor and adapt the network constellations needed for ongoing behaviors, a function rather emerging from an intricate two-way exchange with cortical information than from processing between hierarchically organized brain structures.

## Models of thalamic involvement in linguistic capacities

Various models on the roles of subcortical structures in language processing have been formulated, mostly including the thalamus and the basal ganglia (BG). Closest to the described principles of thalamo-cortical communication is the ‘Selective Engagement Model’ (Crosson, 1985). In this concept, thalamic nuclei, e.g., the centromedian parafascicular complex, monitor the activity state of distributed cortical areas and control their functional connectivity via connections passing through the inferior thalamic peduncle. In case of linguistic information, this primarily refers to frontal and temporo-parietal cortices between which, for example, phonemic, lexical and semantic information is exchanged during language perception and production. In a number of further models, cortico-thalamic language processing is complemented by the BG. These views build upon cortico-striato-thalamo-cortical network properties (Alexander et al., 1986, 1987) the most important of which shall therefore be summarized. In short, the striatum receives excitatory input from almost all cortical regions (Alexander et al., 1986; Mink, 1996; Delong and Wichmann, 2007). Back projection to cortical areas is organized in parallel and interconnected circuitries encompassing limbic, associative and motor loops reaching the BG ‘output nuclei’, namely the internal pallidum (GPi) and the reticular part of the substantia nigra (SNpr) which send inhibiting GABAergic efferences to the thalamus (Alexander et al., 1986; Delong and Wichmann, 2007; Schroll et al., 2012). The ‘output nuclei’ receive convergent signal projections via the ‘direct pathway’ as well as via the ‘indirect pathway’ which encompasses the external pallidum (GPe) and the downstream subthalamic nucleus (STN) (Alexander et al., 1986; Mink, 1996). Concurrently the STN receives ‘hyperdirect’ input from cortical regions (Nambu, 2004; Lambert et al., 2012) which is thought to lead to a procrastination of the BG response (Frank, 2006).

Neurotransmission is mainly GABA-ergic, with the exception of glutamatergic STN output (Wilson, 2004; Delong and Wichmann, 2007). BG activity states are modulated by dopaminergic drive from the pars compacta of the substantia nigra (SNpc) to the striatum (Bamford et al., 2004). For distinct striatal receptor profiles this unfolds complex, partially opposed effects on the direct and indirect route. Finally, the BG output nuclei convey signals to anterior and ventrolateral portions of the thalamus and its intralaminar nuclei and from there back to the cortex (Delong and Wichmann, 2007; Obeso et al., 2008). This assembly of parallel routes with distinct effects on signal propagation, spread and speed is thought to enable the organism with essential functions, such as the selection, temporal filtering and sequencing of competing information (Temel et al., 2005; Tinaz et al., 2006). Further, the mentioned properties have been related to the formation and alignment of behaviors on the basis of habitual rules (Barnes et al., 2005).

In the specific context of language processing, the “Response-Release Semantic Feedback Model” claims thalamic and BG functions in language production (Crosson, 1985, 1992; cf. Murdoch, 2001; Murdoch and Whelan, 2009). As in the Selective Engagement Model, thalamic nuclei are posited to control the interaction between fronto-opercular and temporo-cortical cortices for the integration of lexico-syntactic with semantic information. The resulting signal is further passed on to the BG which are thought to coordinate the release of the provided language plan into speech.

A further dichotomy between thalamic and BG functions in language processing has been suggested in the “Declarative/Procedural Model.” This concept is basically in parallel to claims raised with respect to mnemonic operations (Mishkin et al., 1997; Eichenbaum, 2006). It has been argued that the BG as an apparatus for habit formation and application provide the requirements to apply grammatical rules to linguistic raw data. The supply with respective information, in turn, involves temporo-thalamic networks for transforming arbitrary ‘world knowledge’ into lexical input signals (Ullman, 2001, 2004; Wahl et al., 2008).

Borrowed from the classical model of cortico-striato-thalamo-cortical motor processing (Alexander et al., 1987), the ‘Lexical Selection Model’ views the BG as a machinery to align word-related input to ongoing language plans. This is mainly conceived as a process in which an excess supply of lexical alternatives has to be monitored for unsuited candidate words to be inhibited from further processing. Only the remaining information will be signaled to the thalamus which then initiates fronto-cortical word release (Wallesch and Papagno, 1988; cf. Mink, 1996; Norris and McQueen, 2008).

Finally, it should be mentioned that most linguistic models do not focus on the link between specific operations and brain structures, but rather on the processes themselves. Nonetheless some of these concepts are of interest in this context. In the ‘Motor Theory of Speech Perception’ (Liberman et al., 1967; Liberman and Mattingly, 1985) it is presumed that auditory language information is instantaneously ‘motorically reconstructed’ as an internal imagery of the heard as own speech. Somewhat similarly, the ‘Embodied Cognition Theory’ posits that, e.g., lexical entries are not stored as arbitrary, amodal information, but are tightly linked to sensorimotor processes thought to be necessary for the simulation of word meaning. In line with such ‘horizontal language representation’, it has, for example, been demonstrated that the perception of action words goes along with activations of brain areas involved in the execution of the implied content. Although this refers to the cortical regions (Hauk et al., 2004; Pulvermuller et al., 2005), an additional involvement of basal motor structures could be reasonably assumed for motor imagery and seems supported by respective experiments in patients with BG disease (Tremblay et al., 2008). Future research, particularly in the field of Deep Brain Stimulation (DBS) specifically and reversibly impacting on defined BG structures, might shed light on this open issue, still difficult to access by nowadays research tools.

## Clinical findings

Although, from a clinical perspective, there is little doubt that ‘thalamic aphasia’ exists (Benson and Ardila, 1996; Neau and Bogousslavsky, 1996; Demonet, 1997; Nadeau and Crosson, 1997; Kuljic-Obradovic, 2003; Schmahmann, 2003; De Witte et al., 2011) it is not easy to relate defined language disorders to particular lesions within the thalamus. This is due to a number of reasons. Deficits have often been described in thalamic infarction, but owing to a relatively complex and sometimes variant vascular supply (Van Der Werf et al., 2000; Schmahmann, 2003; Carrera et al., 2004; Carrera and Bogousslavsky, 2006; Hermann et al., 2008; De Witte et al., 2011; Nolte et al., 2011; Hebb and Ojemann, 2012) aphasic syndromes (i) have been observed with different locations of damage, (ii) comprise heterogeneous linguistic deficits, (iii) mostly do not occur in isolation, but go along with other neuropsychological deficits, (iii) and are often accompanied by further extra-thalamic lesions. Further, it has been stated that, in terms of topography, information processed in thalamic areas is propagated in a very divergent and intermingled manner. Hence, the allocation of specific symptoms to particular nuclei is genuinely more difficult at this level than it is for cortical regions with their relatively precise functional description (Bruyn, 1989).

Since language deficits have also frequently been described following other subcortical damage, e.g., BG lesions (Damasio et al., 1982; Perani et al., 1987; Weiller et al., 1990; Hillis et al., 2001; Russmann et al., 2003; Hillis et al., 2004; Han et al., 2005; Choi et al., 2007; Pellizzaro Venti et al., 2012), there have been attempts to differentiate the aphasia types occurring in thalamic vs. striato-capsular and paraventricular white matter infarction (Kuljic-Obradovic, 2003). The authors found that, contrasting these lesion types, thalamic aphasics were characterized by relatively high impairments of comprehension, word finding difficulties and by different types of paraphasias. In other reviews and in-depth studies the scope of language symptoms following thalamic lesions mainly of the dominant hemisphere was relatively wide, the most prominent features being reduced spontaneous speech up to mutism, dysnomia, paraphasias up to jargon and impaired comprehension (Jonas, 1982; Van Der Werf et al., 2000; Schmahmann, 2003; Carrera and Bogousslavsky, 2006; Hermann et al., 2008; De Witte et al., 2011). Many patients had preserved non-propositional speech, i.e., language repetition remained relatively intact. Another striking feature was the preservation of the speech syntax in this cohort. From this it was concluded that a ‘transcortical aphasia’ type was more likely to occur in left thalamic damage than in other brain lesions apart from that of the dominant supplementary motor region (SMR) (Jonas, 1981, 1982). In this regard, thalamic nuclei and the SMR were proposed to form a functional unit in language processing (cf. also Penfield and Roberts, 1959). Further, it should be mentioned that in cases with extended lesions confined to the thalamus, e.g., by primary hemorrhage, permanent global aphasia has been observed (Kumar et al., 1996).

More specific assumptions about the thalamic structures relevant for language processing were formulated by Nadeau and Crosson (Nadeau and Crosson, 1997). Arguments were mainly derived from observations in patients with infarctions of the tuberothalamic and paramedian arteries in which aphasia appears to be more common than after occlusion of the other two thalamic main blood suppliers, the thalamogeniculate and posterolateral choroideal arteries. Particularly tuberothalamic infarction of dependent anterior thalamic structures leads to word finding difficulties, low performance in phonemic and semantic verbal fluency tasks, paraphasia with relatively spared language comprehension. Further, amnesic syndromes may occur and hypomotivational states with apathy, low arousal, and dysexecutive features, in particular, perseverative behavior appear to be common. Although paramedian infarction leads to even more prominent neuropsychiatric sequels (Carrera and Bogousslavsky, 2006), e.g., vigilance fluctuations and confusional states, it can provoke a similar syndrome of transcortical aphasia, comprising reduced word production, phonemic and semantic paraphasia, misnomia and perseverations with largely preserved language comprehension (Molnar, 1959; Davous et al., 1984; Tuszynski and Petito, 1988; Clarke et al., 1994; Van Der Werf et al., 2000; Schmahmann, 2003; Hermann et al., 2008; De Witte et al., 2011). The link between both types of thalamic infarctions was seen in an affection of a network, linking widespread frontal cortex regions with the intralaminar centromedian complex (supplied by the paramedian artery) via the inferior thalamic peduncle, dependent on tuberothalamic supply (cf. Yingling and Skinner, 1977).

Aphasia following infarction of the inferolateral or posterior thalamus, caused by an occlusion of thalamogeniculate or posterolateral choroideal arteries, seems less frequent (Carrera et al., 2004), although it has also been argued that, e.g., the pulvinar had rich collateral perfusion so that respective symptoms might often be compensated (Lhermitte, 1984; Nadeau and Crosson, 1997).

Another line of evidence for thalamic language functions comes from data which were collected in the context of thalamotomy for the treatment of movement disorders (Ojemann and Ward, 1971; Ojemann, 1975, 1976, 1983a, b; cf. Hebb and Ojemann, 2012). In contrast to today, in the nineteen sixties and seventies different target points and trajectories were chosen for this procedure so that cognitive performance could be assessed under test stimulations at different regions of the thalamus. Concerning language, it was found that object anomia and lexical memory disorders followed from current injection in the dorsal lateral thalamus and pulvinar (Fedio and Van Buren, 1975), whereas repetitive (mis)naming was observed under stimulation of the anterior portions of the ventrolateral thalamus and phonemic and syllabic repetitions occurred in intermediate locations. Although from a current perspective, the anatomical precision of these data is not completely clear, they appear to indicate functions of dorsal and, in particular, pulvinar thalamic regions in lexical processing. In this regard, it should be further mentioned that decline in verbal fluency tasks has also been observed in patients with thalamic (DBS) of the ventral intermediate nucleus (VIM) for the treatment of tremor (Troster et al., 1999).

In contrast to the large corpus of evidence that suggests a direct involvement of the left thalamus in language functions, the role of the BG appears more obscure. Clinically, language dysfunctions after BG (capsular/striatal) infarction show high variability and even though aphasia occurs frequently (Weiller et al., 1990; Kumral et al., 1999; Han et al., 2005; Jung et al., 2005; Choi et al., 2007; Pellizzaro Venti et al., 2012) infarction of the BG can also go along with unimpaired language (Nadeau and Crosson, 1997; Kuljic-Obradovic, 2003). Aphasic symptoms are usually distinct from those in ‘thalamic aphasia.’ They have been characterized by widely preserved comprehension, repetition and naming but more strongly affected fluency (Kuljic-Obradovic, 2003). Especially infarction of the caudate nucleus have been associated with perseveration and paraphasia (Kreisler et al., 2000). Causing mechanisms have been discussed controversially, encompassing assumptions about a specific language function of the proper BG (Damasio et al., 1982) and their projections to cortical areas (Perani et al., 1987; Russmann et al., 2003), or alternatively, lesions of white matter fiber pathways (Damasio et al., 1982; Nadeau and Crosson, 1997; Kuljic-Obradovic, 2003), thalamic dysfunction resulting from BG lesions (Pellizzaro Venti et al., 2012), concomitant cortical compression (e.g., of the perisylvian fissure) and cortical hypoperfusion after BG infarction leading to aphasia (Nadeau and Crosson, 1997; Hillis et al., 2001, 2004; Han et al., 2005; Choi et al., 2007).

Apart from aphasic disorders, which thus to many authors do not appear to be a direct cause from BG lesions, stuttering speech is observed after BG lesions (Tani and Sakai, 2011) and can be associated with impaired timing and coordination of language output most likely occasioned directly within the BG (Alm, 2004).

## Experimental findings

At this point, only a small selection of experimental findings on thalamic involvement in linguistic processes shall be given to indicate the heterogeneity of respective data. In a number of functional imaging studies thalamic participation in language tasks has been shown. For example, concerning fundamental functions of language recognition, left thalamic activation has been found during the differentiation of distinct speech sounds, alongside with activity in the planum temporale, the superior temporal and Heschl’s gyri of the dominant hemisphere (Alain et al., 2005). Even the thalamic first-order auditory nucleus, the medial geniculate body, has been shown to be active during the recognition of speech sounds involving cortical feedback loops, a function presumed to be relevant for communication (Von Kriegstein et al., 2008) and which was found impaired in dyslexic persons (Diaz et al., 2012). Further, joint thalamic and frontotemporal involvement has been reported during lexico-semantic operations and object recall (Assaf et al., 2006). Similar functions were allocated to dorsomedial (Van Der Werf et al., 2003) and pulvinar (Nadeau and Crosson, 1997; Kraut et al., 2003a) regions of the thalamus. For the chronometrical sequence of activations during respective tasks, it has been proposed that the dorsomedial thalamus and pre-supplementary motor areas engage in the concept formation of perceived signals, whereas subsequent pulvinar activity reflects downstream processes for the semantic alignment of this with the ongoing input (Kraut et al., 2002, 2003a; Slotnick et al., 2002). The communication with and binding of cortical areas involved in this is thought to be mediated by the pulvinar induction of a common gamma rhythm in, e.g., temporo-parietal regions (Slotnick et al., 2002).

Evidence for cortico-thalamic language processing has also been provided by a study with simultaneous depth and scalp recordings in the context of DBS for the treatment of movement disorders (Wahl et al., 2008). In derivations from DBS electrodes in the ventrolateral thalamus, nearby generated language-related potentials (LRP) were identified during syntactic phrase analysis, interspersed between left frontal and temporo-cortical LRPs. During semantic phrase analysis both cortical and thalamic LRPs appeared as a sustained LRP with indistinguishable dynamics at either level. Of note, during recordings from the relatively close STN no such LRP were seen. Thus, whereas thalamo-cortical networks are involved in complex syntactic and semantic phrase analysis, we did not find evidence that the same held true of the classical cortico-striato-thalamo-cortical circuitry which STN forms part of.

On the other hand, BG contributions to linguistic tasks have indeed been demonstrated. Particularly, the caudate has been found to be involved in a number of linguistic tasks, e.g., concerning word matching operations (Mummery et al., 1998), language selection in bilinguality (Crinion et al., 2006; Friederici, 2006) and lexico-morphological processing (Fiebach et al., 2004) and was supposed to support syntactic operations (Kotz et al., 2009).

On a more general level, a number of studies have assigned rule-based, i.e., grammatical functions of language processing to the BG, as opposed to knowledge-driven, lexical processes. This has been inferred from experimental findings in particular BG disease or under functional manipulation of structures such as the STN by DBS. For example, patients with Chorea Huntington have been reported to display particular difficulties in the analysis of passive instead of active sentence structures which has been interpreted as a striatal deficit of parsing increased grammatical complexity. Further, patients with Parkinson's disease (PD) were found to show increased difficulties in building regular past tense verb forms when their DBS of the STN was switched on vs. switched off (Ullman et al., 1997; Teichmann et al., 2008). In contrast to this, no difference between either stimulation state was obtained for irregular verbs, depending on lexical knowledge instead of rule-based operations exerted on the given verb (Phillips et al., 2012). Thus, a concept for these findings may be that malfunction of the BG leads to deficits in applying combinatorial rules to linguistic messages, compatible with proposed superordinate BG functions, such as the sequencing or time-critical selection of input signals in general (Temel et al., 2005; Tinaz et al., 2006). Another interesting aspect of the above mentioned study was that active vs. inactive DBS of the STN led to enhanced naming of ‘manipulated’ (indicative of a motor connotation) instead of ‘non-manipulated’ (as indicative of absent motor connotations) objects, in parallel with an ameliorated motor condition of PD patients. This appears to tie in with views as formulated in the Embodied Cognition Theory according to which the use and understanding of lexical symbols depends on an internal imagery of their physically experienced implications (see above). In terms of brain structures, such concepts are of interest since they do not imply a strict distinction between sensorimotor and cognitive processes, but rather conceive them as dependent aspects of holistic behavior.

Whichever specific BG structures may be involved in a particular language task, we consider the thalamus - an agglomerate of, admittedly, very distinct nuclear clusters - to contribute to virtually all cognitive demands. This point of view is based on much of the said above, but also on a number of own studies on subcortical processing beyond language. In the aforementioned DBS setting, we addressed functions as diverse as selective attention, response preparedness, cognitive control or the consciousness of perception (Klostermann et al., 2006; Marzinzik et al., 2008; Nikulin et al., 2008; Wahl et al., 2008; Klostermann et al., 2009). We regularly identified neurophysiological correlates of a thalamic involvement, always accompanied by cortically generated event-related potentials (ERP). In the given context, two findings deserve special mention: first, based on chronometrical ERP analysis, thalamic as well as cortical regions could ‘lead’ or ‘initiate’ a given cognitive process (Klostermann et al., 2006); second, in complex ERP sequences the number of components was identical at cortical and thalamic levels, compatible with primary to higher-order event processing in joint subsequent thalamo-cortical steps (Klostermann et al., 2009).

## A concluding view

Coming back to the initial questions of if and how the thalamus contributes to language capacities, we would like to propose (fully aware of the personal angle of this concept) that this is the case in essentially the same way in which it holds true for most non-linguistic cognitive operations. We consider the most relevant thalamic functions to be the control and adaptation of corticocortical connectivity and bandwidth for informational exchange. These functions appear to be built mainly on three properties of thalamocortical neurons: first, their local and remote feed-forward and feed-back connections with almost any cortical region as a prerequisite for establishing flexible network constellations; second, their ability to convey information in distinct discharge modes for regulating the likelihood with which messages are passed on from one cortical region to another, and third, a sequential circuitry of thalamocortical information allowing for the adaptation of final messages in an iterative process involving various downstream relay nuclei.

In this view of transthalamic network orchestration, corticothalamic signals have two functions. They notify thalamic neurons that a specific area has become active, so that functionally related regions can be engaged, for example, the superior temporal gyrus upon fronto-opercular input during word perception. In turn, thalamic neurons will receive the output message from the activated downstream cortex, and so forth. Having said this, it remains open how this iterative process should be adapted to the ongoing demands, particularly in the context of rapidly changing cognitive operations, as required for language processing. In this regard, it is of note that, although thalamic facilitation or disruption of signal propagation has mainly been related to long-acting vigilance conditions, changes of respective neuronal ‘state functions’ certainly also act at shorter intervals in support of ongoing changes of cognitive demands. In terms of language, this can, for example, be conceived as the selection and sequencing of words into phrase structures, emerging from poorly structured phonemic, lexical or syntactic information. On a neuroanatomical level, related functions have been ascribed to the BG, conceived as an apparatus for the coding of overlearned procedures. In this capacity (in line with a number of study results and some clinical observations in subcortical aphasics), the information necessary for the timing of transthalamic cortical engagement could be provided.

Altogether, we favor a concept in which spatially distributed cortical language operations are flexibly (dis-)engaged and (de-)coupled by various thalamic neuronal assemblies, including anterior, intralaminar, dorsomedial nuclei as well as pulvinar subregions of the dominant hemisphere. Further, we consider likely that the timing of transthalamic network constellations is supported by BG computations for the composition of cortically provided information. In this view, human language is not a hierarchically organized cognitive function, but the compound output of interdependent subcortical and cortical systems, specialised in network activation, linguistic programming and temporal process alignment.

### Conflict of interest statement

The authors declare that the research was conducted in the absence of any commercial or financial relationships that could be construed as a potential conflict of interest.
